# Evaluating the impact of female community health volunteer involvement in a postpartum family planning intervention in Nepal: A mixed-methods study at one-year post-intervention

**DOI:** 10.1371/journal.pone.0258834

**Published:** 2021-10-20

**Authors:** Rolina Dhital, Ram Chandra Silwal, Khem Narayan Pokhrel, Sabina Pokhrel, Heera Tuladhar, Suzanna Bright, Emily-Anne Tunnacliffe, Kusum Thapa, Anita Makins

**Affiliations:** 1 Green Tara Nepal, Kathmandu, Nepal; 2 Health Action and Research, Kathmandu, Nepal; 3 Tropical Health and Education Trust, Kathmandu, Nepal; 4 Nepal Society of Obstetricians and Gynecologists, Paropakar Maternity and Women’s Hospital, Kathmandu, Nepal; 5 International Federation of Gynecology and Obstetrics, London, United Kingdom; National University of Singapore, SINGAPORE

## Abstract

**Introduction:**

This is a one-year post-intervention study following an initiative to provide orientation to female community health volunteers (FCHVs) on postpartum family planning in Nepal. In light of positive results in the earlier post-intervention study, this study was designed to provide a more long-term perspective on sustainability by assessing the effect at one-year post-intervention.

**Methods:**

This mixed-methods study was conducted in January 2020 in Morang district, Nepal. We collected quantitative data from a knowledge assessment of FCHVs who had participated in the intervention on postpartum family planning, data on their community-based counseling coverage and through interviews with postpartum mothers in two selected hospitals. Qualitative data were collected through six key informant interviews with health providers and four focus group discussions with FCHVs involved in the intervention. We performed descriptive and multivariate analyses for quantitative data and thematic analysis for qualitative data.

**Results:**

In total, 206 FCHVs participated in the one-year post-intervention study with significant improvement in knowledge of postpartum family planning as compared to pre-intervention period. The adjusted odds ratios (AOR) for knowledge of the 5 key messages on postpartum family planning as compared to the pre-intervention period included 1) knowledge on postpartum family planning can be used immediately after birth (AOR = 18.1, P<0.001), 2) postpartum intra-uterine device (PPIUD) can provide protection up to 12 years (AOR = 2.9, P = 0.011), 3) mothers who undergo cesarean section can use PPIUD (AOR = 2.3, P<0.001), 4) PPIUD can be inserted immediately after birth (AOR = 6.2, P <0.001), and 5) women should go for follow-up immediately if the IUD strings are seen outside vulva (AOR = 2.0, P = 0.08). The FCHVs answering 4 or more questions correctly was 10 times higher (AOR = 10.1, P<0.001) at one-year post-intervention, whereas it was 25 times higher at immediate-post-test (AOR = 25.1, p<0.001) as compared to pre-intervention phase.

The FCHVs had counseled 71% of the pregnant women (n = 538) within their communities at one-year post-intervention. The postpartum mothers in hospitals had a 2 times higher odds of being counseled by FCHVs during their pregnancy at one-year post-intervention (AOR = 1.8, P = 0.039) than in pre-intervention phase. The qualitative findings suggested a positive impression regarding the FCHV’s involvement in postpartum family planning counseling in the communities, however, supervision and monitoring over a longer term was identified as a key challenge and that may influence sustainability of community-based and hospital-based postpartum family planning services.

**Conclusion:**

The FCHVs’ knowledge and community-based activities on postpartum family planning remained higher than in the pre-intervention. However, it declined when compared to the immediate post-intervention period. We propose regular supervision and monitoring of the work of the FCHVs to sustain progress.

## Introduction

In low and middle-income countries such as Nepal, not all women give birth in health facilities, and among those who do deliver in health facilities, return for follow-up remains a challenge [1]. In Nepal, the proportion of women giving birth in health facilities in the presence of skilled birth personnel has increased from 18% to 57% between 2006 and 2016 [2]. Despite the increase in facility deliveries, the utilization of postnatal care services remains low in the country [3].

The barriers to low utilization of postnatal care in Nepal are attributed to socio-cultural factors, difficult geographical and transportation barriers to visiting health facilities in the postnatal period, lack of adequately trained health professionals, and lack of quality care in the health facilities. The low utilization of postnatal care services has also acted as a barrier for uptake of family planning methods during the postpartum period among women in Nepal [3].

Postpartum family planning is the family planning method available for women for the first 12 months of the postpartum period [4]. The family planning methods available after six weeks of childbirth in Nepal for breastfeeding mothers include progesterone-only contraceptives such as oral contraceptive pills, implants, and injectable, non-hormonal methods such as copper intrauterine device (IUD), and female sterilization [5]. The options available for men include condoms and male sterilization throughout the women’s postpartum period [5]. The contraceptive methods available only six weeks after childbirth limit the choices for women who may resume their sexual activities sooner, and the options available for their male partners might not be reliable when women lack autonomy [5,6]. Moreover, for a country with low postnatal care coverage, it limits women’s options for family planning, especially when the women are less likely to return to the facilities [1]. Therefore, postpartum family planning options available immediately after childbirth provide a one-stop solution for those giving birth in the health facilities [1,5,6]. The immediate postpartum family planning services available for women in Nepal include female sterilization and postpartum IUD (PPIUD) [5].

The postpartum family planning initiative was implemented in Nepal jointly by the Nepal Society of Obstetricians and Gynecologists and the International Federation of Gynecology and Obstetrics between 2015 and 2020 [7]. The initiative was primarily hospital-based that focused on training health care providers on the skills of providing immediate postpartum family planning services [7]. The initiative also included hospital-based counseling of pregnant women during their antenatal care (ANC) visits on different postpartum family planning methods [7,8]. However, the awareness and uptake of postpartum family planning methods among postpartum mothers remained lower in Nepal than in other countries [1]. Previous studies from the same initiative identified a lack of community activities as a barrier to the low uptake of immediate postpartum family planning services in Nepal [6,7]. The studies also suggested that female community health volunteers (FCHVs) could play a role in raising awareness about postpartum family planning methods among women in the communities and link the women to the services provided in the hospitals [6,7].

Therefore, an intervention study was designed and implemented as part of the postpartum family planning initiative focusing on FCHVs in Nepal in December 2018 [8]. The goal of the intervention with FCHV was to suffice the existing hospital-based postpartum family planning initiative and link the women in the communities to the hospitals providing postpartum family planning services [8]. The intervention was incorporated into the national FCHV programs and was piloted in Morang district, Nepal. The intervention focused on improving FCHVs’ knowledge of postpartum family planning through an orientation program and supervision of their community activities [8].

The initial intervention study showed that FCHVs had a significant improvement in their knowledge of postpartum family planning as assessed by a post-test on the same day of the training [8]. However, the study lacked a perspective on knowledge retention and the sustainability of the intervention. The follow-up period after two months of the intervention was too short to provide a longitudinal perspective [8]. Therefore, this study aims to assess the effect of the FCHV’s intervention one year after its completion, in terms of FCHV knowledge retention and their community counseling activities in Morang district, Nepal. Comparisons were made with the earlier post-intervention study results [8]. This study could provide a longer-term perspective before expanding FCHVs’ involvement in promoting postpartum family planning at the provincial and national levels in Nepal.

## Methods

This is a one-year post-intervention study that employed a mixed-methods research design. The study followed a sequential explanatory method [9] where the qualitative approach intended to provide insights to the quantitative approach. The Consolidated Criteria for Reporting Qualitative Studies (COREQ) was used to collect, analyze, and report the qualitative findings [10]. The COREQ checklist is provided in the supporting file (S1 Table).

### Study settings

This study was conducted in Morang District, Province One in eastern Nepal. To be in line with the initial post-intervention evaluation [8], this study was conducted at two major referral hospitals in Morang District namely, Koshi Zonal Hospital and Nobel Medical College Teaching Hospital, and the catchment communities of the 23 peripheral health facilities covered by the FCHVs who received the postpartum family planning orientation in December 2018.

The hospitals, peripheral health facilities, and FCHVs represent different layers of the health system in Nepal. The peripheral health facilities represent the primary care centers. However, not all peripheral health facilities are birthing centers and the health providers were not trained on immediate postpartum family methods such as PPIUD at the time of data collection. The peripheral health facilities are linked to the referral hospitals and serve as catchment areas for the hospitals. The two hospitals included in this study were the major referral hospitals in the Morang district providing maternity care and immediate postpartum family planning services [5,8]. Koshi Zonal Hospital is a government hospital and a part of the hospitals implementing the postpartum family planning initiative since 2015. Nobel Medical College Teaching Hospital is a private teaching hospital that started implementing the postpartum family planning initiative in 2018 [5].

### Study participants

#### Quantitative study

The quantitative study participants included FCHVs from the communities and postpartum mothers from the two hospitals. All 230 FCHVs who had participated in the intervention were contacted and invited to participate in the study [8].

The postpartum mothers who had given birth and were admitted at the time of data collection in the two hospitals between January 10, 2020, and February 9, 2020 were considered eligible. We included all women of reproductive age who had delivered at the two hospitals including mothers with newborns who had complications. Mothers with severe maternal postpartum complications, those physically unable to respond to the questions, and those admitted in the intensive care units were excluded.

#### Qualitative study

Qualitative assessment was conducted through key informant interviews (KII) and focus group discussions (FGD). We conducted four FGD with 40 FCHVs who were a subgroup of the 230 FCHVs who had participated in the quantitative study to gain better insights into their knowledge and community-based activities. We purposively selected two facilities with FCHVs having higher knowledge scores and two facilities with FCHVs having lower knowledge scores based on the posttest knowledge scores from previous evaluation study [8]. All the FCHVs approached for FGD agreed to participate.

We also conducted six KII with the stakeholders involved in delivering the intervention for FCHVs. The key informants included:

1. a former director of the Provincial Health Directorate of Province One,
2. a senior public health administrator of Morang district health office who was involved in planning and coordination of the intervention,
3. a doctor and a nurse from the two referral hospitals who were providing postpartum family planning services, and
4. personnel in charge of the health facility from two of the peripheral health facilities supervising FCHVs.

The KII participants were selected purposively based on their active involvement in the intervention.

### Study tools

#### Quantitative tools

For comparison, the same quantitative study tools were used as in the previous post-intervention evaluation study [8]. The tools are provided in the supporting information (S2 and S3 Tables). The tools included:

- **FCHVs’ knowledge questionnaire:** This included five questions which assess knowledge of the key concepts of immediate postpartum family planning and specifically PPIUD (Details in Table 1). The tool was adopted from the government of Nepal’s FCHV User’s Guide on postpartum family planning. The tool was designed to be simple by the government and experts of postpartum family planning in Nepal considering the possibilities of limited literacy of the FCHVs [8].
- **FCHVs’ monthly reporting form:** Information on counseling coverage was collected for a period of two months prior to the start of data collection for this study from November to December, 2019. Indicators included the total number of pregnant mothers in the communities and the proportion of pregnant mothers counseled on postpartum family planning by FCHVs (Table 3).
- **Mothers’ interview questionnaire:** We used the same questionnaire used in previous study [8] to assess if the effect of community counseling coverage by FCHVs was reflected amongst the mothers giving birth in the two referral hospitals. To fulfill the objective of this study, we specifically asked if they had been counseled on postpartum family planning by FCHVs in their community during their pregnancy.

**Table 1:** FCHVs change in knowledge on postpartum family planning.

Knowledge of postpartum family planning	T1	T2	T3	T3-T1	T3-T2
	(n = 230)	(n = 230)	(n = 206)		
**Questions**	n (%)	n (%)	n (%)	(%)	(%)
1. Can mothers use contraception immediately after delivery?	105 (45.7)	173 (75.2)	193 (93.7)	64.0***	0.2***
2. Can Postpartum Intrauterine devices provide protection for up to twelve years?	205 (89.5)	255 (97.8)	198 (96.1)	7.4***	-0.02
3. Can mothers who undergo a caesarean section have postpartum IUD inserted?	115 (50.2)	244 (97.4)	144 (69.9)	39.2***	-28.2***
4. Can IUDs be inserted immediately after giving birth?	148 (64.3)	223 (97.0)	189 (91.7)	17.6***	42.6*
5. Should women go for follow up immediately if the IUD strings are seen outside vulva?	180 (78.3)	215 (93.9)	181 (87.9)	12.3***	-5.6*
**Overall correct answers**					
4 or more correct answers	107 (46.7)	219 (95.6)	186 (90.3)	93.4***	-5.5*

T1 = Pre-test before the orientation, T2 = Post-test immediately after the orientation, T3 = 1-year post-intervention.

*P<0.05.

**P<0.01.

*** P-value<0.001.

### Qualitative tools

- For the qualitative study, the researchers used FGD and KII guides. We used the same FGD guide used in previous study [8] which did not require pretesting. We developed a new KII checklist for this study (S2 Table) which was similar to FGD. Both the tools were reviewed by the qualitative research experts. The guides included the following topics: knowledge of postpartum family planning; impression of the intervention; community-based counseling activities on postpartum family planning; perspectives on the challenges of sustainability; and their suggestions for how to increase long-term impact.

### Study variables

The primary outcomes were FCHVs’ knowledge retention on each of the 5 key messages of postpartum family planning at one year. Other covariates included FCHVs’ age and number of years working as an FCHV. The characteristics of the same population of FCHVs has been described in detail in the previous evaluation study [8].

The secondary outcome was the proportion of women provided counseling on postpartum family planning by FCHVs. The ultimate goal was to prepare and inform the women about the postpartum family planning choices they can use immediately after childbirth. The responses from the postpartum mothers admitted in the hospitals validated the community linkage with the hospitals and reflected the community-based activities conducted by the FCHVs. The exposure variables for the mothers from the two hospitals included the district they came from for delivery and the hospitals where they delivered.

The counseling coverage in the communities was self-reported by FCHVs. The FCHVs’ reports reflected the pregnant women at different stages of pregnancy in the communities in the past two months before data collection.

### Sample size

For FCHVs, we attempted to follow the 230 FCHVs who had participated in the previous evaluation study in pre and post-tests [8]. The details of sampling for the early evaluation has been described in the previous study [8]. A total of 206 FCHVs participated in this one-year post-intervention study, out of the 230 FCHVs with a follow-up rate of 89.6%. Among the 24 FCHVs who did not participate in the follow-up study, two had just retired from their role as FCHV, and 22 did not attend the interview (S1 Fig).

For postpartum mothers, with the assumption that counseling coverage would increase from 7 to 15% between pre and post-intervention, to detect a difference at 80% power and a 5% level of significance [11], a minimum sample of 237 postpartum women was required for one-year follow-up. Allowing for potential incomplete responses a sample size of 300 was adopted with 150 women from each hospital.

### Data collection

#### Quantitative data collection

Quantitative data were collected through face-to-face interviews using smart phones and mobile tablets. The structured data collection tools were developed using Open Data Kit (ODK) software [12]. All the tools were available in both English and Nepali [8]. All study participants were interviewed in Nepali. Data enumerators were trained on data collection techniques, use of the ODK application for smart phones and mobile tablets, ethical considerations, and were provided with an overview of postpartum family planning methods. Data enumerators used the smart phones and mobile tablets to read out the questions to participants and enter their responses.

The 23 peripheral health facilities involved in the training and supervision of the 230 FCHVs coordinated for the data collection. Each facility invited the 9–10 FCHVs who had participated in the intervention within their catchment communities to attend their regular monthly meeting. We chose the same day of the monthly meeting for our data collection at each facility. The FCHVs were provided with travel incentives to attend the monthly meetings as part of the government norms. Data collection for FCHVs took place in January 2020 across 23 peripheral health facilities. All the FCHVs who attended their monthly meeting agreed to participate in the study and gave written informed consent, and were interviewed in a separate room maintaining confidentiality.

For postpartum mothers, we collected the data of the admitted mothers from the two hospitals. We chose the simple random sampling method to recruit the mothers admitted to the two hospitals at the time of data collection. As the healthy mothers and their newborns were discharged within a few days of their childbirth, the enumerators created a sampling frame every day of all the bed numbers of the mothers admitted in the postpartum wards. The enumerators then randomly selected 5 mothers from the list of numbers based on computer-generated random bed numbers for each hospital on each day. The enumerators would request the selected mothers to participate in the study and if they refused, they’d rule out the particular bed numbers and regenerate a new random bed number. The postpartum mothers included in this study represented 30% of an estimated 1000 deliveries over 1 month across the two hospitals. All mothers were provided with an information sheet and their participation was entirely voluntary. Written informed consent was obtained. Mothers were interviewed at their bedside, prior to discharge, without the presence of their caretakers or family members in order to maintain confidentiality.

#### Qualitative data collection

A qualified researcher, independent from the intervention, moderated all the FGD and conducted the KII using the interview checklists. The independent researcher was accompanied by another female researcher who assisted with taking field notes and audio-recording the discussions. Only the two qualitative researchers, and the FCHVs or KII respondents were present in each FGD and KII sessions respectively. The moderator first explained the objectives of the FGD and KII and introduced the research team to the participants. The FGD and KII took around 40 to 45 minutes to complete. The moderator summarized the discussions based on field notes to the FCHVs and stakeholders for their feedback and to ensure that the research team had understood them correctly.

### Ethical considerations

Ethical approval was obtained from Nepal Health Research Council under Ethical Review Board protocol number 823/2019. The written informed consent was collected from all the participants and their privacy and confidentiality were ensured for both quantitative and qualitative studies. Their participation was voluntary and at no point were they pressurized or coerced.

### Data analysis

#### Quantitative analysis

We analyzed three different quantitative datasets using SPSS version 23 with a significance level set at a P-value < 0.05. Bivariate and multivariable analyses were conducted to assess FCHV knowledge retention on postpartum family planning. Chi-squared tests were completed to assess the change in proportions of FCHVs correctly answering questions on each of the five key messages on postpartum family planning (details provided in Table 1), at the post test questionnaire held immediately after the intervention, and one year after the intervention as compared to pre-intervention. Logistic regression models were used to assess the association between the time of the intervention phases and knowledge of each of the five key messages on postpartum family planning, controlling for FCHVs’ age and years of working experience.

Descriptive analysis of counseling coverage by FCHVs in the communities was completed, based on their monthly records of postpartum family planning as reported by FCHVs. Indicators included: total number of pregnant women and the proportion of pregnant women counseled by FCHVs on postpartum family planning.

We compared the proportions of postpartum mothers being counseled by FCHVs during their pregnancy between two-month and one-year follow-up assessments using a chi-squared test.

We then used a multiple logistic regression model to examine the association between the assessment phases and the proportion of mothers reporting to have received postpartum family planning counseling by an FCHV. The logistic regression models controlled for districts the women came from and the hospitals they delivered.

#### Qualitative analysis

Thematic analysis [13] was conducted on the qualitative data based on the five priori themes generated from the two-month post-intervention study [8]. All FGDs were conducted in Nepali and were translated into English language during the process of transcription. The first author and the third author reviewed the transcripts. The first four authors then analyzed the qualitative data using Dedoose software version 8.0.42. The authors first read and re-read the transcripts to familiarize themselves with the broad ideas and generated initial codes. They then gathered all the coded data and collated the codes with the relevant priori themes from the previous study and then selected original quotes from FCHVs and KII for each theme to provide further insight. The first author then compared the responses from the one-year post-intervention study with the responses from the two-month post-intervention study for each theme. The themes were then reviewed by all the authors and assessed to determine whether the data was useful in addressing the overall objective of the study.

## Results

### Quantitative results

Table 1 shows the proportion of FCHVs answering the postpartum family planning questions correctly before the orientation (pre-test), immediately after the orientation (post-test), and one-year after the intervention. A total of 206 FCHVs participated in this one-year post-intervention study, out of the 230 FCHVs who had participated in the intervention at the start (89.6%).

The proportion of FCHVs answering the questions correctly at one-year post-intervention remained significantly higher than pre-intervention for all five questions. However, the proportions of correct answers were lower as compared with the immediate post-test for all but one question.

At one-year post-intervention, the lowest proportion of correct answer was observed for the question on whether a mother who undergoes cesarean section can use a PPIUD. The proportion of FCHVs answering this question correctly decreased by 28% at one-year post-intervention as compared to 39.2% increase in the post-test immediately after the orientation. The percentage of FCHVs answering overall 4 or more questions correctly at one-year post-intervention had decreased by 5.5% than immediate posttest. However, it has increased by 93.4% as compared to pre-test.

Table 2 shows the logistic regression models examining the association between the different phases of assessment of knowledge on each of the 5 key messages of postpartum family planning knowledge among FCHVs. Knowledge of postpartum family planning was also assessed among those with fewer than overall 4 correct answers and those with overall 4 or more correct answers.

**Table 2:** Association between intervention phases and knowledge on key messages of postpartum family planning among FCHVs.

	Knowledge of postpartum family planning		All 3 phases of assessment	
	Characteristics	Assessment	UOR (95% CI)	AOR^a^ (95% CI)
1	Can mothers use contraception immediately after delivery?	Pre-test	1	1
		Post-test	1.4 (0.9–2.1)	3.6 (2.4–5.4)***
		1-year post-intervention	9.7 (5.4–17.6)***	18.1 (9.7–33.6)***
2	Can Postpartum Intrauterine devices provide protection for up to twelve years?	Pre-test	1	1
		Post-test	3.6 (1.4–9.3)**	5.3 (1.9–14.1)**
		1-year post-intervention	1.7 (0.7–3.7)	2.9 (1.3–6.6)**
3	Can mothers who undergo a caesarean section have postpartum IUD inserted?	Pre-test	1	1
		Post-test	25.3 (11.0–58.4)***	37.6 (16.0–88.2)***
		1-year post-intervention	0.82 (0.6–1.2)	2.3 (1.6–3.5)***
4	Can IUDs be inserted immediately after giving birth?	Pre-test	1	1
		Post-test	9.4 (4.3–20.5)***	17.7 (7.9–39.3)***
		1-year post-intervention	2.7 (1.5–4.6)***	6.2 (3.5–10.8)***
**5**	Should women go for follow up immediately if the IUD strings are seen outside vulva?	Pre-test	1	1
		Post-test	3.2 (1.7–5.7)***	4.3 (2.3–7.9)***
		1-year post-intervention	1.7 (0.7–1.9)	2.0 (1.2–3.4)**
	**Overall correct answers**	Pre-test	1	1
** **	4 or more correct answers	Post-test	10.6 (5.5–20.6)***	25.4 (12.6–50.2)***
** **	** **	1-year post-intervention	3.8 (2.3–6.2)***	10.7 (6.3–18.1)***

UOR = unadjusted odds ratio, AOR = adjusted odds ratio.

1 = reference.

NA = not included in the analysis.

* = <0.05

*** = <0.001.

^a^Adjusted for FCHVs’ age and years of experience.

In the adjusted model, a 25-fold increase in FCHV knowledge had been observed at the post-test [AOR = 25.4 (CI 12.6–50.2), P<0.001], and at one-year post-intervention it remained approximately 11 fold higher [AOR = 10.7(CI 6.3–18.1), P<0.001] as compared to the pre-intervention phase.

The knowledge for each of the five key questions had significantly improved as compared to the pre-intervention phase. The knowledge on postpartum family planning can be used immediately after birth was 18 folds higher (AOR = 18.1, P<0.001) in one-year post intervention which was higher than the increase in knowledge at immediate post-test (AOR = 3.6, P<0.001) as compared to the pre-test before the intervention. The knowledge on PPIUD can provide protection for up to 12 years was 3 folds higher at one-year post-intervention (AOR = 2.9, P = 0.011) as compared to pre-test, whereas it had increased by 5 folds at immediate post-test (AOR = 5.3, P = 0.001). The knowledge on mothers who undergo a cesarean section can have PPIUD inserted had retained at 2 folds higher (AOR = 2.3, P<0.001) at one-year post-intervention, whereas it had increased by 38 folds at immediate post-test (AOR = 37.6, P<0.001). The knowledge on PPIUD can be inserted immediately after birth had retained at 6 folds higher at one-year post-intervention (AOR = 6.2, P<0.001) as compared to 18 folds higher at immediate post-test (AOR = 17.7, P<0.001). The knowledge on women should go for follow-up when the IUD strings are seen outside of vulva was retained by 2 folds higher at one-year post-intervention (AOR = 2.0, P = 0.08) whereas it had increased by 4 folds at immediate post-test (AOR = 4.3, P<0.001).

Table 3 shows the descriptive data collected from the peripheral health facilities of the monthly meeting records collected and submitted by FCHVs, on their community-based activities. It shows the service coverage for a period of two months before the data was collected for each phase—at two-months post-intervention and at one-year post-intervention. At two-months post-intervention, all the 23 peripheral health facilities had maintained monthly records of postpartum family planning service coverage by FCHVs [8]. At one-year post-intervention, 13 out of 23 facilities had maintained the monthly reporting forms. At one-year post-intervention, the proportion of pregnant women counseled by FCHVs was 71.5% (n = 538) as compared to 83.3% (n = 1559) at two-months post-intervention.

**Table 3:** Proportion of pregnant mothers counseled by FCHVs in the communities and uptake of PPIUD in facilities.

Description	2-months post-intervention	1-year post-intervention
No. peripheral health facilities maintaining FCHV records	23	13
No. recorded pregnant mothers in the catchment areas of FCHVs	1872	752
Proportion of all the recorded pregnant mothers counseled by an FCHV on postpartum family planning	1559 (83.3%)	538 (71.5%)

**Fig 1:**
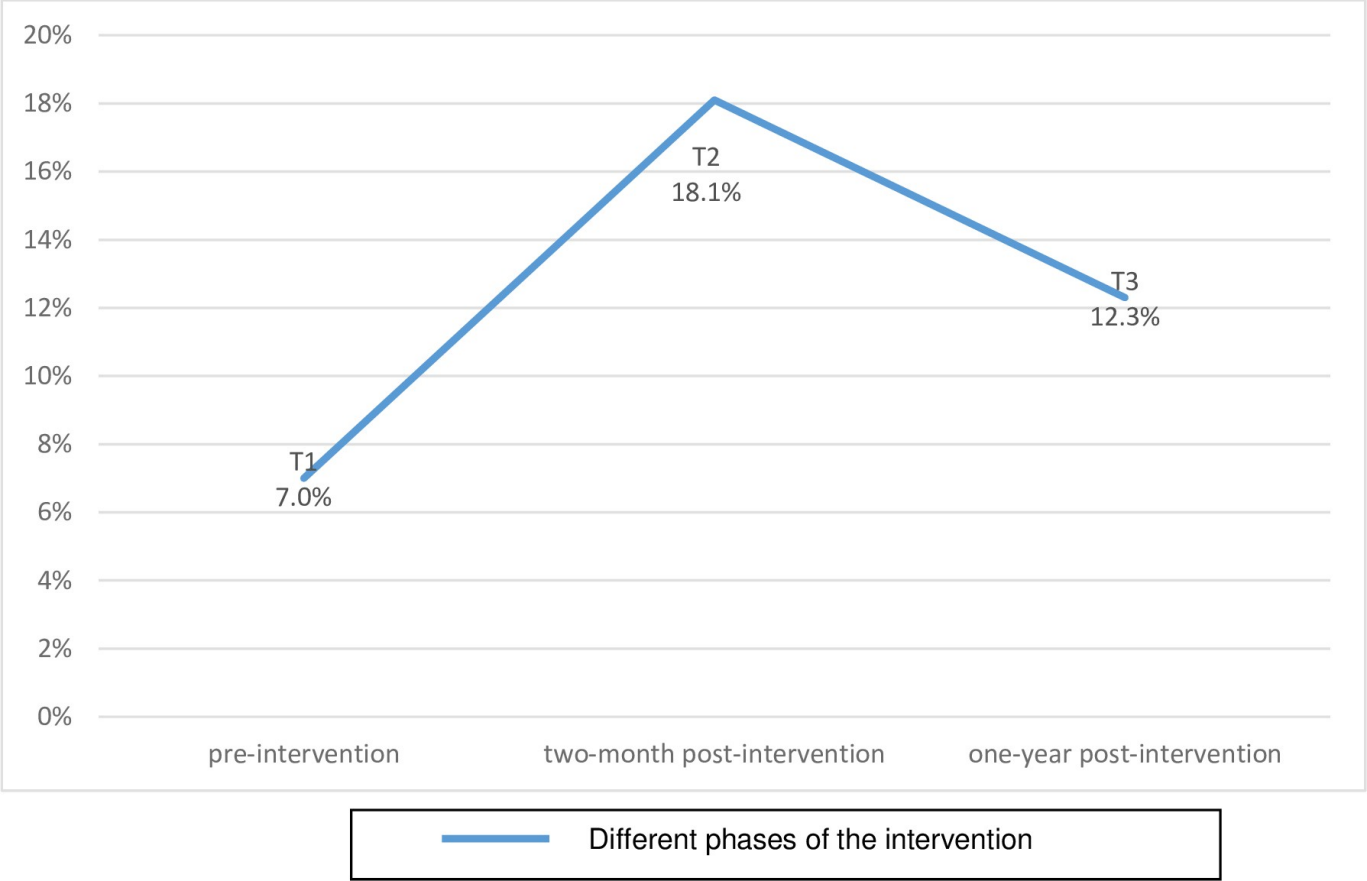
Counseling coverage of PPFP among the postpartum mothers by FCHVs in the two referral hospitals. (T1) pre-intervention, (T2) two-month post-intervention, (T3) one-year post-intervention.

Fig 1 shows the proportion of postpartum mothers in the hospitals who were counseled by an FCHV during their pregnancy in the community. In total, data was collected from 244 women in the pre-intervention phase, 238 at two-months post-intervention and 300 at one-year post-intervention.

The proportion of women that reported they were counseled by FCHVs at one-year post-intervention was 12.3% (n = 37) of 300 women, which was higher than the pre-intervention phase of 7% (n = 17) of 244 women. However, it was lower than 18.1% (n = 43) of 238 women interviewed at two-months post-intervention (Fig 1).

Table 4 demonstrates the logistic regression model examining the relationship between time period from intervention and the number of postpartum mothers reporting receiving counseling from an FCHV during their pregnancy.

**Table 4:** Association between intervention phases and FCHV counseling among postpartum mothers delivering in the two hospitals.

	Counselled by FCHV	
Characteristics	UOR (95% CI)	AOR (95% CI)
**Assessment**		
Pre-intervention	1	1
2-month post-intervention	2.0 (1.3–3.1)**	2.9 (1.6–5.4)***
1-year post-intervention	0.9 (0.6–1.5)	1.9 (1.0–3.5)*
**Facility**		
Nobel Medical College teaching hospital	1	1
Koshi Zonal Hospital	1.5 (1.0–2.4)*	1.5 (0.9–2.5)
**District**		
Other	1	1
Morang	1.1 (0.7–1.7)	1.01 (0.6–1.6)

UOR = unadjusted odds ratio.

AOR = adjusted odds ratio.

1 = reference.

NA = not included in the analysis.

*** = <0.001.

* = <0.05.

** = <0.01.

In the adjusted model, there was an almost 3-fold increase [AOR = 2.9, (CI 1.6–5.4), P<0.001] in the number of women reporting being counseled by an FCHV at two-months post-intervention, and an almost 2-fold increase [AOR = 1.9, (1.0–3.5), P = 0.036] at one-year post-intervention, when comparing to the pre-intervention phase.

### Qualitative results

Table 5 summarizes the key themes of the qualitative study comparing the findings between the two-month post-intervention evaluation study, and the one-year post-intervention study. These include the FGD findings for the two-month post-intervention, and FGD and KII findings for one-year post-intervention. There were no KII in the earlier study. Comparisons were made on the five key themes which included knowledge of postpartum family planning, perception on the postpartum family planning orientation, activities conducted by FCHVs in the communities, challenges of maintaining their work, and suggestions to improve sustainability. Original quotes from the responses of the participants at one-year post-intervention are included in the description of the findings to provide more context.

**Table 5:** Comparison between two-month and one-year post-intervention qualitative findings.

	Themes	2-months post-intervention FGD	1-year post-intervention FGD and KII
**1**	**Knowledge on postpartum family planning **	• Majority of FCHVs were able to mention timing of insertion and benefits correctly.	• Majority of FCHVs were able to mention timing of insertion and benefits correctly.
		• Some FCHVs were confused about whether PPIUD can be used among women who had undergone the Cesarean section.	• The confusion regarding PPIUD use among women who underwent Cesarean section persisted. • Some FCHVs were also confused about follow-up among PPIUD users and lacked knowledge about referring women with PPIUD strings seen or felt outside the vulva to the health facilities.
**2**	**Perception of postpartum family planning orientation program**	• The majority of FCHVs considered the postpartum family planning program to be useful.	• The majority of FCHVs and stakeholders still considered postpartum family planning program useful.
**3**	**Activities conducted by FCHVs on postpartum family planning**	• Almost all the FCHVs had actively conducted activities in the communities and raised awareness about postpartum family planning in healthy mothers’ groups and counseled pregnant women in the communities. • All FCHVs had actively maintained their monthly reporting forms.	• Not all FCHVs were actively involved in counseling activities for pregnant women in the communities. • Many FCHVs had stopped maintaining the monthly FCHV reporting forms citing lack of supervision and support.
**4**	**Challenges of sustaining their work**	• FCHVs were concerned about the potential threats they may have to face while counseling women in the communities. • Many FCHVs considered women to face societal barriers to choosing postpartum family planning/PPIUD	• No threats were encountered by FCHVs in their community-based activities since the 2-month study. • Many FCHVs still considered societal barriers to exist, preventing women from choosing postpartum family planning/PPIUD • FCHVs suggested that a change of new peripheral ‘facility in-charge’ may have interrupted the regular monitoring of FCHV activities related to postpartum family planning services. • Stakeholders highlighted concern regarding a lack of refresher courses and monitoring.
**5**	**FCHVs’ suggestions on improving sustainability**	• Strong request for additional refresher courses for their knowledge retention.	• Many FCHVs and KII highlighted the need for refresher courses and better monitoring and supervision of FCHV related activities. • Stakeholders highlighted the need for local greater government involvement for sustainability.

#### 1. Knowledge of postpartum family planning

At one year, almost all FCHVs still considered postpartum family planning as a new concept. Most were able to list out different methods of postpartum family planning and also explain the time of insertion of PPIUD as well as its benefits, as had been true at the two-months post-intervention study [8].

*“In my knowledge*, *PPIUD is a convenient method to be inserted in uterus of a recently delivered woman*. *This protects a woman from unwanted pregnancy*. *The merit of this method in my view is that one does not need to wait until the next menstrual cycle after childbirth*.*”-*FCHV, FGD 1-year post-intervention

Some FCHVs lacked adequate knowledge about certain aspects of PPIUD at one year. During the orientation, FCHVs were taught that the uterus involutes over time and therefore, it is important to refer the PPIUD users to the hospital for follow-up as well as to cut the thread when it is seen outside the vulva. Contrary to this, some FCHVs considered having a string seen or coming out of the vulva, to be normal.

*“When the thread/string is seen or felt by the women coming out of vulva outside the body*, *it is normal*. *They don’t need to worry about it or go for follow-up to the hospital*.*”-*FCHV, FGD 1-year post-intervention

*Perception about postpartum family planning orientation*. FCHVs during the two-month post-intervention study suggested that the orientation had helped to improve their postpartum family planning knowledge [8] and at one-year post-intervention, all FCHVs from the FGD and stakeholders from KII still considered this to be the case. All participants believed that efforts in continuing the orientation activities must be expanded and refresher orientations should be initiated to maintain the momentum of FCHV led community-based activities on postpartum family planning.

*“It was one of the best trainings that I have ever had*! *The contents were understandable*, *trainers motivated everyone to learn*, *and overall I liked the training session*.*”-*FCHV, FGD 1-year post-intervention

*“There is no doubt that FCHV initiated counseling is effective in terms of finding women in ANC and discussing their need for the PPIUD*. *Training helped them by motivating and identifying new knowledge regarding the method and the need to counsel pregnant women in their communities*.*”-* Public Health Administrator, Health Office, Morang, KII

#### 2. Activities conducted by FCHVs in the communities

In the two-month post-intervention study all FCHVs appeared to be motivated and had shared their postpartum family planning related activities in the communities enthusiastically, however at a one-year post-intervention, activities appeared to have slowed down. All the FCHVs at two-month post-intervention mentioned that they had been maintaining the FCHV monthly reporting forms on postpartum family planning. Whereas, at one-year post-intervention, almost half of the FCHVs acknowledged that they had stopped maintaining the monthly reporting forms. They cited discontinuation of supervision from those in charge of the health facilities and a lack of reporting forms since the start of the new fiscal year (mid-July, 2019) as some of the key reasons for their discontinuation. A few, mentioned that they still counsel women about postpartum family planning methods as needed even though they had stopped maintaining a record.

Major changes in the stakeholders working at different levels of the health system had occurred since the last evaluation. More than 50% of the peripheral health facilities had newly assigned health facility ‘in-charges’ and staff. Whilst most of the peripheral health facilities which had retained the same people in charge, were able to continue monitoring the FCHV activities on postpartum family planning, those with newly assigned supervisors (who were not involved in the initial intervention) were unaware of the FCHV activities regarding postpartum family planning and had therefore not been monitoring their activities. It seemed that there was little monitoring of activities at a central level.

*“Projects come and go*. *So we thought it’s the same with this one too*. *When the project is active everybody is active*. *Once it phases out no one really asks us to report it either*. *Our new health facility-in-charge too stopped asking about the data*. *So we discontinued filling the monthly data for postpartum family planning*.*”-*FCHV, FGD 1-year post-intervention

#### 3. Challenges of sustaining their work

In the two-month post-intervention research, the key challenges expressed by the FCHVs included societal barriers for women to accept immediate postpartum family planning methods such as PPIUD and the fear of potential threats from people in the communities when providing counseling. At one-year post-intervention, most of the participants still cited the low acceptability of PPIUD due to societal barriers. However, none of the FCHVs had faced challenges from the communities, allaying their earlier fears.

One of the major challenges indicated by most of the FCHVs and KII participants at one year was the lack of monitoring and supervision since the intervention ended. Some FCHVs also highlighted the gap in linking their counseling to the actual services women would receive in the two hospitals.

The stakeholders from the two hospitals highlighted that while FCHVs involvement in the communities is useful, they also felt that designated counselors are needed in the hospitals to help bridge the gap and to supplement community-based counseling services.

*“Counseling of the pregnant women in the communities is very useful and it supplements the counseling services in the hospital*. *However*, *the women also need thorough counseling again when they come to the hospital*. *Due to high workload*, *not all the trained providers in the hospital are able to provide counseling*…*”-*Nursing in-Charge-Hospital, KII

Other challenges highlighted by FCHVs and stakeholders alike included a lack of clarity in the referral of mothers for postpartum family planning services and a lack of postpartum family planning services in lower level facilities.

*“Sometimes when we counsel pregnant mothers it’s difficult for us to decide where to advise them to go for delivery*. *It would have been easier if the postpartum family planning/PPIUD facilities were available in nearby places*. *Not all women are ready going to the two big hospitals for delivery and the place of their choice does not have those facilities*.*”-*FCHV, FGD 1-year post-intervention

Former director of the Provincial Health Directorate, who had been recently transferred to work at central level in Kathmandu, indicated additional challenges for sustainability such as: the short implementation phase of FCHV involvement in the project; inadequate exit strategy for FCHV activities after the project ended; and a lack of involvement of birthing centers who could also have provided postpartum family planning services.

“*Involving FCHVs for postpartum family planning was the first project of its kind for the government too*. *But the duration of implementation was too short*. *Such projects require a minimum period of one or two years of implementation to be sustainable*. *But this project ended without a detailed exit strategy*.”-Former Director of Provincial Health Directorate of Province 1, KII

#### 4. Suggestions on sustainability

In the two-month post-intervention study, FCHVs had requested a refresher course. At one-year post-intervention, both FCHVs and other stakeholders highlighted that refresher orientation for FCHVs had not taken place and continued to highlight the importance of such ongoing orientation. Many FCHVs also suggested that having additional counseling materials such as flipcharts on postpartum family planning and sample IUDs would help their counseling to be more effective.

*“There has been no follow-up or refresher orientation for FCHVs*. *It would be better if they could receive such refresher course in every 6 months*. *It would also have been helpful if the (government) health office would follow-up and monitor the FCHV activities on a regular basis even though the project has ended*.*”-*health facility-in-charge, KII

Almost all FCHVs focused on the need for follow-up of activities, which ended after the completion of the project. Many FCHVs and many stakeholders also urged for an expansion of postpartum family planning services into nearby health facilities for increased sustainability and equity of service access.

*“For sustainability*, *services for PPIUD should be provided in our nearby health facility as the majority of our clients come from poor and unreached backgrounds and not everyone goes to those two hospitals where postpartum family planning services are provided*.*”-*FCHV, FGD 1-year post-intervention

## Discussion

The intervention in this study assessed the knowledge retention of FCHVs about postpartum family planning so that they could counsel the women in the communities and link them to the hospitals providing immediate postpartum family planning services [8]. Retained knowledge at one-year post-intervention among FCHVs in this study was higher as compared to the pre-intervention period which is encouraging for this cadre of community health workers who do not have a professional degree in health sciences. However, knowledge had decreased when compared to the earlier evaluation highlighting the challenges of sustainability. For FCHVs to retain knowledge in the long-term, refresher trainings would be advisable.

A review of the existing literature on long-term retention of knowledge suggests that 66 to 75% of knowledge will be retained after one year in general education as well as in medical education [14]. The FCHVs in this study had a higher retention rate than this. However, knowledge is likely to decrease further by 50% or more in the next five years without any further ongoing training or education [14]. Another review of ongoing trainings for community health workers from various low- and middle-income countries on different health topics has shown positive results for knowledge retention following refresher trainings [15]. A study on refresher training for community health workers in India suggested the knowledge retained among them following the refresher training at one-year post-intervention was higher than the knowledge retained two months after the initial training [16]. Thus, it would be important for the local and provincial government to identify strategies to conduct refresher courses and regular supervision to help the FCHVs retain their knowledge of postpartum family planning.

The secondary goal of the FCHV intervention was to suffice the existing postpartum family planning counseling services in the hospitals and establish a community linkage with the hospitals. In this study, the proportion of women counseled by FCHVs remained higher than the pre-intervention period for women giving birth in the two hospitals, however, no improvement was observed as compared to two-months post-intervention period. Lack of improvement in the proportion of women being counseled as compared to early post-intervention phase reflect the challenges of sustaining the intervention and inadequate linkage between FCHV’s community activities with the hospitals. The two hospitals included in this study were implementing hospital-based interventions on postpartum family planning services as part of the larger postpartum family planning initiative in Nepal. The unadjusted logistic regression model suggested that the postpartum mothers in Koshi Zonal Hospital were 1.5 times more likely to have been counseled by FCHVs during their pregnancy in the communities than the women from Nobel Medical college. Koshi Zonal Hospital is a major referral government hospital in Morang district and therefore, is directly linked to the government peripheral health facilities and community-based activities by FCHVs [7]. Moreover, the hospital had been implementing the postpartum family planning initiative since 2015 [7]. Likewise, Nobel Medical College Teaching Hospital is a private teaching hospital that had started implementing the postpartum family planning initiative only in 2018 [5]. As a private hospital, the linkage with the FCHV’s community-level activities might not have been as strong as a government hospital would have. However, previous study on service coverage from the same initiative had indicated a better hospital-based counseling coverage and acceptance of postpartum family planning methods in the private hospital which could balance the weaker community linkage to some extent [5].

The descriptive data collected from the FCHVs’ monthly reporting forms on their community-based postpartum family planning counseling coverage showed incomplete recording and reporting of their activities. FCHVs conduct health awareness programs every month within the catchment areas of their peripheral health facilities through monthly mother’s group meetings [8]. They also counsel pregnant women in the communities on birth preparedness, identify danger signs, and encourage pregnant women to go to health facilities for childbirth [17,18]. They are provided with a monthly reporting book by the government and use it as a tool to record their activities and report it to the peripheral health facilities towards the end of every month. As part of the intervention for postpartum family planning, an additional simple checklist was introduced by the implementers of the intervention [8]. However, the new checklist has not yet been incorporated within the national system for FCHV’s monthly reporting book. This could have been one of the reasons behind some FCHVs not continuing recording and reporting activities for postpartum family planning.

Moreover, the qualitative findings also indicated that many peripheral health facilities had newly assigned health facility in-charges who were unaware of the intervention and didn’t supervise the FCHVs’ postpartum family planning related activities. At the time of this study, we identified that more than 50% of the peripheral health facilities had newly assigned health facility-in-charges who were not part of the intervention. The changes in the peripheral health personnel has been part of the larger reshuffling process taking place in the health system in Nepal [19]. Although there are no studies looking into the effects of this reshuffling on the sustainability of health programs; it could have interrupted the recording and reporting activities among FCHVs. It is postulated that the new staff may not have recorded activities accurately and therefore the actual counseling of expectant mothers by FCHVs could have been under-reported. Given the retention in their knowledge at one year, particularly regarding the value of immediate postpartum family planning, it is not unreasonable to assume that counseling would have continued albeit un-recorded. Moreover, the trained health personnel who had moved on to new facilities may have helped in training new FCHVs who were not part of this intervention. Future follow-up studies focusing on the activities of providers who moved to new facilities could provide an interesting perspective on sustainability.

The qualitative findings of this study showed that FCHVs and the stakeholders regarded PPIUD as the most useful postpartum family planning method and believed that the FCHV activities must be expanded. They also considered that the orientation of FCHVs was a useful component of the intervention and their continued involvement would support hospital-based postpartum family planning services. However, it was also highlighted that the lack of refresher orientation for FCHVs and inconsistent monitoring of their activities after one-year was problematic for sustainability. The World Health Organization recommends the need for ongoing training activities with regular supervision and refresher training for community health workers [4]. The lack of consistent supervision of the activities among FCHVs identified in this study is also likely to affect sustainability of the intervention in years to come. The ongoing training activities have also been regarded as a neglected aspect of most training programs for community health workers in low-and middle-income countries [20]. The United States Agency for International Development Health Care Improvement Project has recommended that community health workers should be updated every six months from their initial training to sustain the practice of their skills [21].

The previous studies from the initiative had identified the need of coordinated efforts from different levels of health system and society to bring positive behavior changes related to acceptance of postpartum family planning [6,7] FCHVs represent the community level and their role was to counsel the women in the communities about the immediate postpartum family planning choices they will have in hospitals [8]. However, FCHVs are not the direct service providers and are only a dimension of the larger intervention [1,6,8]. Thus, their role in acceptance of PPIUD and other postpartum contraception among women would remain limited. Therefore, the acceptance of PPIUD or other contraceptives were not considered as an outcome for FCHV intervention on postpartum family planning.

The ultimate goal of this intervention was to improve the acceptance of immediate postpartum family planning methods through a coordinated effort involving different tiers of the health system in Nepal including FCHVs. FCHVs have played an important role in mobilizing the communities and strengthening the maternal and newborn health and family planning coverage in Nepal [22–24]. Globally, community health workers have also played crucial roles in improving the knowledge, attitude, and uptake of modern contraceptives in general [25]. However, the intervention studies on training community health workers to improve immediate postpartum family planning acceptance and uptake remain scant. The intervention involving FCHVs for postpartum family planning was the first of its kind in Nepal. The findings on knowledge retention on postpartum family planning at one-year post-intervention are encouraging. However, further studies are warranted to assess the specific roles of FCHV in improving the acceptance and uptake of immediate postpartum family planning methods such as PPIUD.

### Policy implications

Community health workers all around the world have a unique role to play in health care, as they are not government employees and yet are often expected to undertake specific health counseling roles which in many high-income countries would be the remit of salaried, formally trained nurses or midwives. Perhaps one of the issues with community health workers is that they fall into a vacuum of responsibility–because they are not officially recognized salaried government staff, they are often trained and supported by the government tasked with filling in the gap. This can result in a fragmented approach to their training and function. This is unfortunate, given they are highly respected by their communities and so hugely influential, particularly in remote communities such as rural Nepal and therefore could be instrumental in changing the behavior of communities. One suggestion could be to recognize the value of this cadre of health personnel by remunerating their work appropriately, providing regular training and mentoring as well as monitoring their outputs.

The continued efforts of FCHVs was reflected amongst the mothers giving birth in the two major referral hospitals. At one-year post-intervention, the odds of a postpartum mother in one of the hospitals having been counseled by an FCHV during their pregnancy remained two times higher as compared to the pre-intervention period. Moreover, the knowledge retention of postpartum family planning among FCHVs at one-year follow-up and their continued efforts with community-based counseling have both remained higher than in the pre-intervention phase. Despite the lack of continued supervision, the activities are still functioning which is remarkable. However, in order to sustain the progress, the suggestions from the study participants must be taken into account such as the need to provide refresher orientations for the FCHVs and providing orientations for newly assigned health facility personnel. Timely acknowledgement of such potential barriers by the concerned policymakers of Nepal is essential. Further, lobbying for the continuation of refresher courses for FCHVs, ensuring better monitoring, recording, and reporting of postpartum family planning activities to enable data-driven decision making, and incorporating postpartum family planning activities into the national FCHV program are necessary next steps to ensure sustainability of postpartum family planning activities already embarked upon. Moreover, efforts in coordinating between different layers of health system and strengthening the community linkages with the hospitals is essential to improve women’s access and utilization to the services provided in the higher level hospitals. Follow-up studies to evaluate the intervention after a longer period are also necessary to assess the progress and sustainability.

### Limitations

This study has certain limitations regarding its study design. It has no control group, which could have provided a comparative perspective on postpartum family planning services in areas without any intervention. However, it would not have been feasible to recruit control groups from the same setting as other FCHVs beyond the intervention catchment area had also received orientations on postpartum family planning at some point and the spill-over effect of the intervention couldn’t be ruled out. Due to feasibility issues, it was also not possible to recruit FCHVs from other districts.

Secondly, the recording and reporting of the postpartum family planning counseling activities by FCHVs may not reflect the true picture of the actual counseling activities by FCHVs. Thus, the under-reporting of the postpartum family planning activities is highly likely given the fact that the correct forms were not issued and there was a change of supervisory staff at many peripheral health facilities. Interviewing the women or potential clients of FCHVs could have provided a more direct perspective from the clients’ point of view. However, it was beyond the scope of our study as the primary contacts of the FCHVs are women in the communities for which we would have had to do a more extensive community-based household survey. However, we did assess a subset of the women from the community who had gone to the selected two facilities for childbirth. A wider community-based survey in the future could help reflect the sustainability in the communities in the long-term.

Thirdly, the tool we used for assessing FCHV’s knowledge was not able to assess experiences and awareness at a deeper level. However, FCHVs in Nepal mostly have limited literacy and henceforth we had to use a simple questionnaire which was a validated tool used by the government as part of their training guideline. Moreover, qualitative research complemented the quantitative study where the study participants were able to share experience, awareness, and technical understanding.

Fourthly, as a longitudinal study, regression errors could have been affected by repeat observations. It would have been ideal to perform statistical analyses with models such as generalized estimating equation modeling to minimize the errors for repeated observations. However, the participants were not assigned the same identification numbers for each assessment which was a limitation during data collection.

Fifthly, the odds ratio was quite large with relatively wide confidence intervals when the follow-up results were compared with the baseline results for FCHVs’ knowledge. Though the increase in knowledge after the intervention was remarkable, the possibility of sparse data bias leading to a large odds ratio and wide confidence intervals cannot be completely ruled out.

Lastly, the findings of qualitative studies at two months and one year after the interventions provide a perspective of changes taking place but may not be directly comparable. The people interviewed are different and the time the interviews took place is also different. Similarly, KII was not undertaken in the first 2 months’ post-intervention study and so comparisons are not like for like.

This study nevertheless, provided a longitudinal perspective on the changes taking place over time. Moreover, as a mixed-methods study, the qualitative findings helped to provide more context to the results obtained from the quantitative study.

### Conclusion

This study showed that the knowledge of postpartum family planning among FCHVs and their counseling activities remained higher at a one-year follow-up as compared to the pre-intervention phase. However, it had decreased as compared to the earlier evaluation highlighting the challenges of sustainability. The proportion of women being counseled by FCHVs remained higher than in pre-intervention phase and no significant difference was observed between two- month post-intervention and one-year post-intervention assessments. Continued supervision and monitoring was identified as a way to maintain postpartum family planning activities amongst FCHVs and refresher trainings would likely help in maintaining knowledge and sustaining progress in the long-term. In view of these findings, incorporating postpartum family planning activities into the national FCHV program would be strongly advised.

## Supporting information

S1 Fig Timeline of the postpartum family planning initiative and the intervention. (DOCX)

S1 Table COREQ checklist. (PDF)

S2 Table Study tools in Nepali. (PDF)

S3 Table Study tools English. (DOCX)
