# Supplementary material for: Evaluating the impact of female community health volunteer involvement in a postpartum family planning intervention in Nepal: A mixed-methods study at one-year post-intervention
Source: PLoS One. 2021 Oct 20;16(10):e0258834. doi: 10.1371/journal.pone.0258834 (PMC8528303; doi:10.1371/journal.pone.0258834)
Supplement: S1 Fig — (DOCX) [file pone.0258834.s001.docx]

**S4 Fig. Timeline and the flow of activities for postpartum family planning initiative and FCHV intervention**

206 FCHVs participated in the one-year post-intervention study.

(2 retired, 22 did not attend the interview for data collection)

Cascade process to deliver the intervention to FCHV

Year

**2018-2019**

**2020**

**2019**

**2015**

One-year post-intervention study

Early evaluation study of the FCHV research

230 FCHVS participated in the intervention and early evaluation study

Each peripheral facility trained and supervised 9-10 FCHVs

15 Provincial level facilitators

92 Health care providers from 23 peripheral facilities

FCHV Intervention added to existing interventions in Morang district

Postpartum Family Planning Initiative implemented in Nepal

Hospital-based
