# Supplementary material for: Evaluating the impact of female community health volunteer involvement in a postpartum family planning intervention in Nepal: A mixed-methods study at one-year post-intervention
Source: PLoS One. 2021 Oct 20;16(10):e0258834. doi: 10.1371/journal.pone.0258834 (PMC8528303; doi:10.1371/journal.pone.0258834)
Supplement: S2 Table — (PDF) [file pone.0258834.s003.pdf]

महिला स्वास्थ्य स्वयम सेविका(FCHV)अन्तर्वार्ताको प्रश्नावलीहरु

(स्वास्थ्य सेवा प्रदान गर्नेले भर्ने)

अन्तवार्तामिति :.................................अन्तवार्ता लिनेको नाम:...

स्वास्थ्य सेवा संस्था को नाम ....

महिला स्वास्थ्य स्वयम सेविकाको नाम :..

साधारण जानकारी

	प्रश्नहरु	उत्तरहरु
१.	तपाई कति बर्षको हुनुभयो?	.... वर्ष
२.	तपाईले कति कक्षा सम्म पढ्नुभएको छ?	(क) पढ्न र लेख्न सक्छु ।<br>(ख) प्राथमिकशिक्षा (कक्षा १ देखि ८ सम्म)<br>(ग) माध्यमिकशिक्षा (कक्षा ८ देखि १३ सम्म)<br>(घ) विश्वविद्यालयशिक्षा (स्नातकशिक्षा र माथि)
३.	तपाईले महिला स्वास्थ्य स्वयम सेविकाको रुपमा काम गरेको कति समय भयो ?	.... वर्ष
४.	गएको एक वर्षमा तपाईंले समुदायको महिलाहरु लाई सुत्केरिमा प्रयोग गरिने परिवार नियोजनको साधन सम्बन्धि परामर्श दिनु भएको छ?	(क) छ<br>(ख) छैन

प्रसुति पछि प्रयोग गरिने परिवार नियोजन सम्बन्धि/आई.यु.डी (PPIUD) सम्बन्धि सम्बन्धि ज्ञान

	प्रश्नहरु	उत्तरहरु
१.	बच्चाजन्मनेबित्तिकै महिलाले परिवार नियोजनको साधनप्रयोग गर्नु हुन्छ।	(क) ठीक ☺ (ख) बेठीक ☹
२.	बच्चाजन्मनेबित्तिकै प्रयोग गरिने परिवार नियोजनको साधन आई.यु.डी (कपर-टि) ले १२ बर्षसम्मकामगर्दछ ।	(क) ठीक ☺ (ख) बेठीक ☹
३.	शल्यकृयाद्धारा बच्चा जन्मिएपछि पाठेघरमा राख्न सकिन्छ	(क) ठीक ☺ (ख) बेठीक ☹
४.	बच्चाजन्मिनेबितिकै पाठेघरमा आई.यु.डी (कपर-टि) राख्न सकिन्छ	(क) ठीक ☺ (ख) बेठीक ☹
५.	योनी बाहिर आई.यु.डी (कपर-टि) धागो देखिएमा नजिकको स्वास्थ्य केन्द्रमा जाँचको लागि जानुपर्दछ ।	(क) ठीक ☺ (ख) बेठीक ☹

Interview Questionnaire for postpartum mothers

सुत्केरी आमा हरु को लागि अन्तर्वार्ता प्रश्नावली

Registration

INTRODUCTION by the DCOs to the participant

1. क्रम संख्या – (ID number generated in the app)
2. अस्पतालको नाम

   1. कोशी अञ्चल अस्पताल

   2. नोबेल मेडिकल कलेज टिचिंग अस्पताल

3. तथ्याङ्क सङ्कलन अधिकृतको नम्बर..
4. अन्तर्वार्ता मिति ………………………………
5. प्रसुति भएको (बच्चा जन्मेको) मिति …………………………..

Part I General Information

Questions

	Question	Response	Remarks
1.	तपाईं हाल कता बस्नु हुन्छ ?<br><br>*(बस्दै आएको मुख्य ठाउँ)*	1. प्रदेश …………….<br>2. जिल्ला …………..<br>3. वडा …………….<br>4. पालिका …………	Text<br>Fill each
•	तपाईं कुन धर्म मान्नु हुन्छ?	1. हिन्दु<br>2. बौध्ध<br>3. मुस्लिम<br>4. क्रिस्चियन<br>5. अन्य	Multiple choice<br>(select one)
•	तपाईं कुन जाति मा पर्नु हुन्छ ?	1. ब्रह्मिन छेत्री<br>2. जनजाती	Multiple choice

		3. दलित<br>4. मधेसी<br>5. अन्य	(select one)
•	तपाईं ले कति पढ्नु भएको छ ? (*पुरा भएको पढाइ)*	1. पढ्न र लेख्न आउदैन<br>2. पढ्न लेख्न आउछ<br>3. प्राथमिक तह (कक्षा १ देखि ८ सम्म)<br>4. माध्यमिक तह सम्म (कक्षा ९ देखि १२ सम्म)<br>5. विश्व बिद्यालय तह (स्नातक वा सो भन्दा बढी<br>6. अन्य (जस्तै धार्मिक शिक्षा -मदरसा आदि)	Multiple choice (select one)
•	तपाइंको हाल बैवाहिक अवस्था कस्तो छ ?	1. श्रीमान संगै<br>2. श्रीमान संग छुट्टिएको<br>3. बिधवा<br>4. अन्य	
•	तपाईं श्रीमान संगै हुनु हुन्छ भने, वहाँ ले कति पढ्नु भएको छ ?<br>(*पुरा भएको पढाइ)*<br>Skip the question if not currently married such as separated/divorced/widow	1. पढ्न र लेख्न आउदैन<br>2. पढ्न लेख्न आउछ<br>3. प्राथमिक तह (कक्षा १ देखि ८ सम्म)<br>4. माध्यमिक तह सम्म (कक्षा ९ देखि १२ सम्म)<br>5. विश्व बिद्यालय तह (स्नातक वा सो भन्दा बढी<br>6. अन्य (जस्तै धार्मिक शिक्षा -मदरसा आदि)	Multiple choice (select one)

Part II Pregnancy and childbirth

	**Question**	**Response**	**Remarks**
•	तपाइँ ले यसपटक को समेत गरेर कति पटक जन्म दिनु भएको छ ?<br>(parity)	………………… पटक	numerical
•	तपाइंको हाल बच्चाको अवस्था कस्तो छ ?	1. जिउदो<br>2. मरेको	
•	तपाईंको अहिले को बच्चा को लिङ्ग के हो ? Skip if the newborn is dead	1. केटि<br>2. केटा<br>3. अन्य	

•	अन्तिम गर्भावस्था (यस पटक को बच्चा गर्भ मा हुदा) मा तपाईं ले गर्भ जांच गर्नु भएको थियो ?	1. थियो<br>2. थिएन	
•	अन्तिम गर्भावस्था (यस पटक को बच्चा गर्भ मा हुदा) मा तपाईंले यहि अस्पतालमा गर्भ जांच गर्नु भएको थियो ?	1. थियो<br>2. थिएन	
•	तपाईंको प्रसुति कसरी (कुन मध्यम बाट) भाको थियो ?	1. यौनि बाट (vaginal)<br>2. अपरेशन बाट (cesarean)	Choose 1

Part III FCHV related

13.	अन्तिम गर्भावस्था (यस पटक को बच्चा गर्भ मा हुदा) मा तपाईंको महिला स्वयम् सेविका संग कहिले अंतर्किर्या (भेटघाट) भएको थियो ?	1. थियो<br>2. थिएन	Choose 1
14.	यदि अंतर्किर्या (भेटघाट) भएको थियो भने कुन स्वास्थ्य चौकीमा भएको थियो ?	1. झोराहट<br>2. बैजनाथपुर<br>3. मोतिपुर<br>4. कटहरी<br>5. मझारे<br>6. पथरी<br>7. मंगलबारे<br>8. हत्तिमोड़ा<br>9. टंकी<br>10. रानी<br>11. इन्द्रपुर<br>12. दुलारी	13. मृगौलिया<br>14. सुन्दरपुर<br>15. सोराभाग<br>16. दादर्बेरिया<br>17. भातिगंज<br>18. बुद्धनगर<br>19. जहदा<br>20. लखन्तरी<br>21. हरिचा<br>22. कसेनी<br>23. भौडा<br>24. अन्य - नाम खुलाउनु होस्
15.	तपाईंले अन्तिम गर्भावस्थामा महिला स्वयम् सेवकी संग अंतर्किर्या (भेटघाट कुराकानी) गर्दा, वहाँ हरुले तपाईंलाई प्रसुति पछि प्रयोग गरिने परिवार नियोजन सम्बन्धि परामर्श (सल्लाह सुझाब) दिनु भएको थियो ?<br><br>Skip if never interacted with FCHV	1. थियो<br>2. थिएन	
16.	तपाईंले अन्तिम गर्भावस्थामा महिला स्वयम् सेवकी संग अंतर्किर्या (भेटघाट कुराकानी) गर्दा, वहाँ हरुले तपाईंलाई	1. थियो<br>2. थिएन	

	प्रसुति पछि प्रयोग गरिने आई यु डी (कपर-टी) भन्ने परिवार नियोजन सम्बन्धि परामर्श (सल्लाह सुझाब) दिनु भएको थियो<br><br>Skip if never interacted with FCHV		
17.	महिला स्वयम् सेविका ले तपाईंलाई प्रसुति पछि प्रयोग गरिने परिवार नियोजन सम्बन्धि थप जानकारी लिन अस्पताल वा स्वाथ्य संस्थान जानु भनेर सल्लाह दिनु भएको थियो ?<br><br>Skip if never interacted with FCHV	1. थियो<br>2. थिएन	

Part IV PPFP/PPIUD counseling coverage

18.	यस अस्पतालमा तपाईंलाई विभिन्न प्रकारका प्रसुति पछि प्रयोग गरिने परिवार नियोजन्को साधन सम्बन्धि परामर्श गरेको थियो ?	1. थियो<br>2. थिएन	Choose one
19.	यस अस्पतालमा प्रसुति पछि प्रयोग गरिने आई यु डी (कपर-टी) PPIUD भन्ने परिवार नियोजन सम्बन्धि परामर्श सम्बन्धि परामर्श गरेको थियो ?	1. थियो<br>2. थिएन	
20.	यदि यस अस्पतालमा तपाईंलाई परामर्श दिएको थियो भने, कुन बेला परामर्श दिएको थियो ?<br><br>Skip if never counseled about PPIUCD in this hospital	1. गर्भावस्था जांचको बेला<br>2. प्रसुति भए पछि<br>3. गर्भावस्था जांच र प्रसुति पछि दुबै पटक	Choose one

Part V PPFP/PPIUCD uptake

21.	के तपाईंले अहिले प्रसुति पछि प्रयोग गर्ने आई यु डी (कपर-टी) प्रयोग गरिराख्नु भएको छ ?	1. छ<br>2. छैन	Choose one
22.	यदि प्रयोग गरिराख्नु भएको छ भने स्वास्थ्य प्रदायक ले (डाक्टर वा नर्सले) कुन बेला पाठेघर भित्र हाली दिनु भएको थियों ? Skip if currently not using PPIUCD	1. प्रसुति भएको तुरुन्तै (बच्चा जन्मेर साल निस्के पछि) (Postplacental)<br>2. बच्चा जन्माउने पेटको अपरेसन भैराखेको बेला (intracesarean)<br>3. प्रसुति पछि वार्ड मा सरिसके पछि (post-partum)	Choose one

23.	यदि प्रसुति पछि प्रयोग गर्ने आई यु डी (कपर-टी) (PPIUD) प्रयोग गर्नु भएको छैन भने, यहि बच्चा जन्मेको एक वर्ष भित्र अन्य कुनै प्रसुति पछि प्रयोग गरिने परिवार नियोजनको साधन प्रयोग गर्न इच्छुक हुनु हुन्छ ?<br><br>Skip if currently using PPIUCD	1. छ<br>2. छैन	Choose one
24.	यदि इच्छुक हुनु हुन्छ भने कुन परिवार नियोजनको बिधि प्रयोग गर्ने इच्छा छ ?<br><br>(Skip if currently using PPIUCD<br><br>OR<br><br>Do not wish to use any method)	1. प्राकृतिक बिधि<br>2. पिल्स चक्की<br>3. कन्डम<br>4. डिपो<br>5. इम्प्लान्ट<br>6. आइ यु डी<br>7. थाई बिधि (पुरुष वा महिला बन्ध्याकरण)<br>8. अन्य	Choose one Preferred the most

FGD checklists for FCHVs

- Introduction by the research team
- Introduction of the FCHVs

Overall perception about PPFP/PPIUD

• प्रसुति पछि प्रयोग गरिने परिवार नियोजन सम्बन्धि उपायहरुको बारेमा तपाईं लाई कतिको जानकारी छ?
• के कस्ता उपायहरु थाहा छ तपाइंलाई? प्रकारहरुको नाम भनि दिनु हुन्छ?
• प्रसुति पछि प्रयोग गरिने आई.यु.डी (कपर -टी) को बारेमा कतिको जानकारी छ?
• प्रसुति पछि प्रयोग गरिने आई.यु.डी (कपर -टी) को बारे तपाईंको कस्तो धारणा छ?
• पोहोर सालको अभिमुखी कार्यक्रम पछि तपाइंको प्रसुति पछि प्रयोग गरिने आई।यु।डी (कपर -टी) को बारेसोचाइ / धारणामा केहि परिवर्तन आएको छ?
-यदि आएको छ भने के-कस्तो प्रकारको परिवर्तन आएको छ?
-यदि आएको छैन भने किन आएन?

Perception about PPFP orientation program

• पोहोर सालको अभिमुखी कार्यक्रम तपाईंलाई कस्तो लागेको थियो?
• त्यस अभिमुखी कार्यक्रमका के के कुरा तपाईंलाई मन परेको थियो?
• त्यस अभिमुखी कार्यक्रमका के के कुरा तपाईंलाई मन परेको थिएन?
• अभिमुखी कार्यक्रम पछि प्रसुति पछि प्रयोग गरिने परिवार नियोजन सम्बन्धि मुख्य के के कुरा सिक्नु भयो जस्तो लाग्छ?
• अभिमुखी कार्यक्रम पछि प्रसुति पछि प्रयोग गरिने आई.यु.डी (कपर -टी) को सम्बन्धि मुख्य के के कुरा सिक्नु भयो जस्तो लाग्छ?

PPFP counseling and referral behavior

• अभिमुखी कार्यक्रममा सहभागी हुनु अघि तपाईंले कुनै महिला लाई प्रसुति पछि प्रयोग गरिने परिवार नियोजन सम्बन्धि परामर्श दिनु भएको थियो?<br>-यदि थियो भने के कस्तो सल्लाह सुझाब दिनु भएको थियो?
• अभिमुखी कार्यक्रममा सहभागी हुनु भए पछि तपाईंले कुनै महिला लाई प्रसुति पछि प्रयोग गरिने परिवार नियोजन सम्बन्धि परामर्श दिनु भएको थियो?<br>- यदि थियो भने के कस्तो सल्लाह सुझाब दिनु भएको थियो?
• प्रसुति पछि प्रयोग गरिने आई यु डी (कपर-टी) बारे कुनै महिलालाई परामर्श दिनु भएको छ ?<br>- यदि थियो भने के कस्तो सल्लाह सुझाब दिनु भएको थियो?
• कुनै महिला लाई प्रसुति पछि प्रयोग गरिने आई यु डी (कपर-टी) प्रयोग गर्न अस्पताल पठाउनु/ प्रेषण गर्नु भएको छ?<br>-यदि छ भने कसरि प्रेषण गर्नु भयो?<br>-यदि छैन भने किन गर्नु भएन?
• प्रसुति पछि प्रयोग गरिने आई यु डी (कपर-टी) प्रयोग गरेको महिला मा कुनै प्रकार को समस्या देखा परेको भेट्नु भएको छ?<br>-यदि छ भने कस्तो प्रकारको समस्या देखा परेको थियो?<br>-त्यो समस्या को समाधान गर्न तपाइंले के गर्नु भयो?
• समुदायमा महिला हरुलाई परामर्श गर्दा तपाइंले के कस्ता कठिनाई हरु भोग्नु परेको छ? (तपाईंको कुनै बिशेष अनुभव भनिदिनु हुन्छ?)

- तपाइंले प्रसुति पछि प्रयोग गरिने परिवार नियोजन सम्बन्धि परामर्श दिई सक्नु भए पछि तपाईंको समुदायमा सकारात्मक परिवर्तन हरु के के देख्नु भयो? (तपाईंको कुनै बिशेष अनुभव भनिदिनु हुन्छ?)

Recommendations

- महिला स्वयम् सेविका लाई दिने प्रसुति पछि को परिवार नियोजन सम्बन्धि अभिमुखी कार्यक्रम लाई थप सुधार गर्न के कस्तो सल्लाह सुझाब दिन चाहनु हुन्छ?
  अरु कुनै थप सल्लाह सुझाब दिन चाहनु हुन्छ?

Note: further questions will be added based on any specific findings from the quantitative study, to explain the quantitative findings better

KII checklists for stakeholder

- Introduction by the research team

- Introduction of the KII

Overall perception about PPFP/PPIUD

• प्रसुति पछि प्रयोग गरिने परिवार नियोजन सम्बन्धि नेपालमा भैरहेको कार्यक्रम हरु प्रति तपाईंको कस्तो धारणा छ ?
• प्रसुति पछि प्रयोग गरिने आई.यु.डी (PPIUD) सम्बन्धि नेपालमा भैरहेको कार्यक्रम हरु प्रति तपाईंको कस्तो धारणा छ ?
• प्रसुति पछि प्रयोग गरिने परिवार नियोजन सम्बन्धि कार्यक्रममा महिला स्वास्थ्य स्वयम् सेविकालाई यस मोरंग जिल्लामा संलग्न गराइएकोमा तपाइंको के कस्तो धारणा छ ?
• के तपाइंलाई महिला स्वयम् सेविकाको अभिमुखी कार्यक्रम पश्चात उनीहरुको प्रसुति पछि प्रयोग गरिने परिवार नियोजन सम्बन्धि धारणा परिवर्तन भएको छ जस्तो लाग्छ ?
-यदि छ भने, के कस्तो परिवर्तन भएको छ जस्तो लाग्छ ?
-यदि छैन भने किन परिवर्तन भएन जस्तो लाग्छ ?

Perception about PPFP orientation program

• पोहोर सालको महिला स्वास्थ्य स्वयम् सेविकाको अभिमुखी कार्यक्रम तपाईंलाई कस्तो लागेको थियो ?
• त्यस अभिमुखी कार्यक्रमका के के कुरा तपाईंलाई मन परेको थियो ?
• त्यस अभिमुखी कार्यक्रमका के के कुरा तपाईंलाई मन परेको थिएन ?
• अभिमुखी कार्यक्रम पछि प्रसुति पछि प्रयोग गरिने परिवार नियोजन सम्बन्धि महिला स्वास्थ्य स्वयम् सेविका हरुले मुख्य के के कुरा सिक्नु भयो जस्तो लाग्छ ?
• अभिमुखी कार्यक्रम पछि प्रसुति पछि प्रयोग गरिने आई.यु.डी (कपर -टी) को नियोजन सम्बन्धि महिला स्वास्थ्य स्वयम् सेविका हरुले मुख्य के के कुरा सिक्नु भयो जस्तो लाग्छ ?

PPFP counseling and referral behavior

• अभिमुखी कार्यक्रममा सहभागी हुनु अघि महिला स्वास्थ्य स्वयम् सेविकाले आफ्नो समुदायमा महिला हरुलाई प्रसुति पछि को परिवार नियोजन सम्बन्धि परामर्श दिनु भयो जस्तो लाग्छ?<br>- यदि लाग्छ भने, के कस्तो सल्लाह सुझाब दिनु भयो जस्तो लाग्छ?<br>-तपाइंले कुनै प्रतक्ष देख्नु भएको छ? वा सुन्नु भएको छ?
• अभिमुखी कार्यक्रममा सहभागी हुनु भए पछि महिला स्वास्थ्य स्वयम् सेविका ले आफ्नो समुदायमा महिला हरुलाई प्रसुति पछि को परिवार नियोजन सम्बन्धि परामर्श दिनु भयो जस्तो लाग्छ?<br>- यदि लाग्छ भने, के कस्तो सल्लाह सुझाब दिनु भयो जस्तो लाग्छ?<br>-तपाइंले कुनै प्रतक्ष देख्नु भएको छ? वा सुन्नु भएको छ
• प्रसुति पछि प्रयोग गरिने आई यु डी (कपर-टी) बारे महिला स्वास्थ्य स्वयम् सेविका ले कुनै महिलालाई परामर्श दिनु भएको छ जस्तो लाग्छ ?<br>- यदि थियो भने के कस्तो सल्लाह सुझाब दिनु भएको थियो जस्तो लाग्छ?<br>-तपाइंले कुनै प्रतक्ष देख्नु भएको छ? वा सुन्नु भएको छ
• कुनै महिला लाई प्रसुति पछि प्रयोग गरिने आई यु डी (कपर-टी) प्रयोग गर्न महिला स्वास्थ्य स्वयम् सेविका ले अस्पताल पठाउनु/ प्रेषण गर्नु भएको छ ?

	-यदि छ भने कसरि प्रेषण गर्नु भयो जस्तो लाग्छ?<br>-यदि छैन भने किन गर्नु भएन जस्तो लाग्छ?
	• प्रसुति पछि प्रयोग गरिने आई यु डी (कपर-टी) प्रयोग गरेको महिला मा कुनै प्रकार को समस्या देखा परेको भेट्नु भएको छ ?<br>-यदि छ भने कस्तो प्रकारको समस्या देखा परेको थियो ?<br>-त्यो समस्या को समाधान गर्न तपाइंले के गर्नु भयो ?
	• समुदायमा महिलाहरु लाई परामर्श गर्दा महिला स्वास्थ्य स्वयम् सेविका ले के कस्ता कठिनाई हरु भोग्नु परेको छ ? (तपाईंको कुनै बिशेष अनुभव भनिदिनु हुन्छ ?)
	• महिला स्वास्थ्य स्वयम् सेविकाले प्रसुति पछि प्रयोग गरिने परिवार नियोजन सम्बन्धि परामर्श दिई सक्नु भए पछि तपाईंको समुदायमा सकारात्मक परिवर्तन हरु के के देख्नु भयो? (तपाईंको कुनै बिशेष अनुभव भनिदिनु हुन्छ?)

Sustainability Recommendations

	के तपाइंलाई महिला स्वयम् सेविकाले सामुदायिक परामर्श गर्नु प्रसुति पछि को परिवार नोयोजन कार्यक्रम को लागि एक दिगो समाधान हो जस्तो लाग्छ ?<br>-यदि लाग्छ भने किन?<br>-यदि लाग्दैन भने किन ?
	• महिला स्वयम् सेविका लाई दिने प्रसुति पछि को परिवार नियोजन सम्बन्धि अभिमुखी कार्यक्रम लाई थप सुधार गर्न के कस्तो सल्लाह सुझाब दिन चाहनु हुन्छ ?<br><br>• अरु कुनै थप सल्लाह सुझाब दिन चाहनु हुन्छ ?

Note: further questions will be added based on any specific findings from the quantitative study, to explain the quantitative findings better

Checklist for FCHVs monthly reporting forms

FCHV ID NO……….

Name of the health facility…………….

	Shrawan	Bhadra	Asoj	Kartik	Mangsir
Number of pregnant women in the community					
Number of women delivered in the community					
Number of women counseled on PPFP					
Number of women using PPIUD					

Checklist of PPFP uptake in peripheral health facility

Name of the health facility…………..

Note: data should include the mothers in postpartum period only within 12 months of childbirth

	**Shrawan**	**Bhadra**	**Asoj**	**Kartik**	**Mangsir**
**PPFP types**					
PPIUD Insertion					
Female sterilization					
Male Sterilization					
Interval IUD					
Implant					
Male condom					
Injectable					
Oral contraceptive pills					
