# Supplementary material for: Evaluating the impact of female community health volunteer involvement in a postpartum family planning intervention in Nepal: A mixed-methods study at one-year post-intervention
Source: PLoS One. 2021 Oct 20;16(10):e0258834. doi: 10.1371/journal.pone.0258834 (PMC8528303; doi:10.1371/journal.pone.0258834)
Supplement: S3 Table — (DOCX) [file pone.0258834.s004.docx]

**S2 Table Study tools (English version)**

**Interview Questionnaire for Female Community Health Volunteers**

**Interview date:…………………………… Interview by:………………………………**

**Name of the health facility………………….**

**Name of the FCHV…………………..**

**General information**

|  | **Questions** | **Responses** |
| --- | --- | --- |
|  | How old are you? | …………………..yrs |
|  | What is your highest level of education? | 1. Cannot read and write 2. Can read and write 3. Primary level (grade 1 to 8) 4. Secondary level (grade 8 to 12) 5. University level (bachelors degree and above) |
|  | How long have you been working as FCHV? | …………..yrs |
|  | In the past one year have you ever counselled women in your community about postpartum family planning | 1. Yes 2. No |

**Knowledge on PPFP/ PPIUCD**

|  | **Questions** | **Responses** |
| --- | --- | --- |
|  | Immediately after delivery, women can use contraception | 1. True 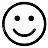 2. False 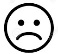 |
|  | Postpartum Intrauterine devices can provide protection up to twelve years | 1. True 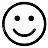 2. False 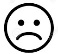 |
|  | Women who undergo a caesarean section can have postpartum IUCD inserted | 1. True 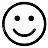 2. False 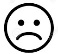 |
|  | IUCDs can be inserted immediately after giving birth | 1. True 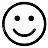 2. False 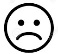 |
|  | If IUCD strings are seen outside vagina, they should go for follow up immediately | 1. True 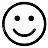 2. False 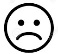 |

**Interview Questionnaire**

**Registration**

**INTRODUCTION by the DCOs to the participant**

- - 1. Respondent ID Generation – (ID number generated in the app)
    2. Facility Name
       1. Koshi Zonal Hospital, Biratnagar
       2. Nobel Medical College Teaching Hospital
    3. DCO number * *(an identification number is provided for each DCO)……………………..*
    4. Date of Interview………………………………
    5. Date of Delivery …………………………..

**Part I General Information**

**Questions**

|  | Question | Response | Remarks |
| --- | --- | --- | --- |
|  | Where do you live currently  *(The main place of residence during pregnancy and childbirth)?* | 1. District……………. 2. Pallika…………… 3. Ward No………….. | Text  Fill each |
|  | What is your religion? | 1. Hindu 2. Budhist 3. Christian 4. Muslim 5. Others | Multiple choice  (select one) |
|  | What is your ethnicity? | 1. Brahmin/Chetri 2. Janajati 3. Dalit 4. Madhesi 5. Others | Multiple choice  (select one) |
|  | What is your education level?  *(completed highest level of education)* | - - - 1. Cannot read and write       2. Can read and write only       3. Primary level (grade1 to 8)       4. Secondary level (grade 9 to 12)       5. University level (bachelors and above)       6. Others (such as religious schools) | Multiple choice  (select one) |
|  | What is your current marital status? | 1. With husband 2. Separated/divorced 3. Widow 4. others |  |
|  | What is your husband’s level of education?  *(completed highest level of education)*  Skip the question if not currently married such as separated/divorced/widow | - - - 1. Cannot read and write       2. Can read and write       3. Primary level (upto grade 8)       4. Secondary level (grade 9 to 12)       5. University level (bachelors and above)       6. Others (such as religious schools) | Multiple choice  (select one) |

**Part II Pregnancy and childbirth**

|  | **Question** | **Response** | **Remarks** |
| --- | --- | --- | --- |
|  | How many times have you given birth including the current childbirth? | …………………times | numerical |
|  | What is the status of your current newborn? | 1. Alive 2. Dead |  |
|  | What is the gender of your current newborn? (if alive)  Skip if the newborn is dead | 1. Male 2. Female 3. Others |  |
|  | Did you attend ANC for this (last) pregnancy? | 1. Yes 2. No |  |
|  | Have you ever attended ANC in this hospital for this (last) pregnancy? | 1. Yes 2. No |  |
|  | What was the mode of your delivery? | 1. Vaginal 2. Cesarean section | Choose 1 |

**Part III FCHV related**

| 13. | Have you ever interacted with an FCHV during your last pregnancy? | 1. Yes 2. No | Choose 1 |
| --- | --- | --- | --- |
| 14. | If yes, where did you meet/interacted with the FCHV? | 1. Jhorahat PHC 2. Baijhanathpur HP 3. Motipur HP 4. Katahari HP 5. Majhare HP 6. Pathari HP 7. Manglbare 8. Hattimoda HP 9. Tanki HP 10. Rani PHC 11. Indrapur HP 12. Dulari HP | 13. Mrigauliya HP  14. Sundarpur HP  15. Sorabhag HP  16. Dadarberiya HP  17. Bhatigunj HP  18. Buddhanagar HP  19. Jahada HP  20. Lakhantari HP  21. Haricha PHC  22. Kaseni HP  23. Bhaudha HP  24. Others- Specify |
| 15. | Did the FCHV counseled you about different methods of PPFP in your last pregnancy?  Skip if never interacted with FCHV | 1. Yes 2. No |  |
| 16. | Did the FCHV counseled you about PPIUD in your last pregnancy? Skip if never interacted with FCHV | 1. Yes 2. No |  |
| 17. | Did the FCHV suggested you to go to the health facility to learn more about PPIUD? Skip if never interacted with FCHV | 1. Yes 2. No |  |

**Part IV PPFP/PPIUD counseling coverage**

| 18. | Were you ever counseled about different methods of PPFP in this hospital? | 1. Yes 2. No | Choose one |
| --- | --- | --- | --- |
| 19. | Were you ever counseled about PPIUD in this hospital? | 1. Yes 2. No |  |
| 20. | If you were counseled on PPIUD in this hospital, when were your counseled?  Skip if never counseled about PPIUCD in this hospital | 1. ANC 2. After childbirth 3. Both during ANC and after childbirth | Choose one |

**Part V PPFP/PPIUCD uptake**

| 21. | Are you currently using PPIUD? | 1. Yes 2. No | Choose one |
| --- | --- | --- | --- |
| 22. | If using PPIUCD, when was it inserted by the provider?  Skip if currently not using PPIUCD | - - 1. Immediately after delivery in labour room (Postplacental)     2. Immediate after delivery in operating room (Intracesarean)     3. After being transferred to the ward (post-partum) | Choose one |
| 23. | If not using PPIUCD, do you plan to use any other methods of PPFP within one year?  Skip if currently using PPIUCD | 1. Yes 2. No | Choose one |
| 24. | If you plan to use other PPFP methods within one year, which method would you prefer? | 1. Natural methods 2. OCP 3. Condoms 4. Depot 5. Implant 6. Interval IUCD 7. Permanent methods 8. Others | Choose one  Preferred the most |

**KII checklists for stakeholder**

- Introduction by the research team
- Introduction of the KII

**Overall perception about PPFP/PPIUD**

| - What is your perception of PPFP programs in Nepal? |
| --- |
| - What is your perception about PPIUD? |
| - What is your perception of involving FCHV in PPFP program in Morang district? |
| - Do you think the perception about PPFP and PPIUD changed after the orientation program among FCHVs and in the communtiies? |
| -If yes, why and what are the changes in your perceptions? |
| -If No, why? |

**Perception about PPFP orientation program**

| - How did you find the orientation program? |
| --- |
| - What you were the things you liked about the orientation program? |
| - What were the things you did not like about the orientation program? |
| - What were the key messages you think FCHVs learned about PPFP after the orientation program? |
| - What were the key message that you FCHVs learned about PPIUD after the orientation program? |

**PPFP counseling and referral behavior**

| - Do you think FCHVs had ever counseled any women about PPFP before they attended the orientation?   -If yes, what kind of suggestions they might have given?  (any example you have witnessed or ware of)? |
| --- |
| - Do you think FCHVs have counseled any women about PPFP after they attended the orientation?   -If yes, what kind of suggestions they might have given?  (any example you have witnessed or ware of)? |
| - Do you think FCHVs have explained specifically about PPIUD to any women?   -If yes, what they might have explained  (any example you have witnessed or ware of)? |
| - Do you think FCHVs have referred anyone to hospital to use PPIUD?   If yes, why? And how they might have refered?  If No, why?  (any example you have witnessed or ware of)? |
| - Did you come across any women with complications or problems with PPIUD?   If yes, what were the types of complications or problems you found?  And did you or the health facility do to overcome the problem? |
| - What are the challenges that you think FCHVs might have faced while counseling women in the communities about PPFP? (could please share your story, if any) - What are the positive changes you noticed in your community after FCHV’s counseling activities? (could you please share your story, if any) |

**Sustainability Recommendations**

| Do you think involving FCHV for community counseling on PPFP is a sustainable solution?  If yes, why  If no, why |
| --- |
| What are the recommendations you would like to give to the concerned stakeholders to improve such orientation programs in the future? |

**Note: further questions will be added based on any specific findings from the quantitative study, to explain the quantitative findings better**

**Checklist for FCHVs monthly reporting forms**

**FCHV ID NO………..**

**Name of the health facility…………….**

|  | **Shrawan** | **Bhadra** | **Asoj** | **Kartik** | **Mangsir** |
| --- | --- | --- | --- | --- | --- |
|  |  |  |  |  |  |
| **Number of pregnant women in the community** |  |  |  |  |  |
| **Number of women delivered in the community** |  |  |  |  |  |
| **Number of women counseled on PPFP** |  |  |  |  |  |
| **Number of women using PPIUD** |  |  |  |  |  |

**Checklist of PPFP uptake in peripheral health facility**

Name of the health facility……………

Note: data should include the mothers in postpartum period only within 12 months of childbirth

|  | **Shrawan** | **Bhadra** | **Asoj** | **Kartik** | **Mangsir** |
| --- | --- | --- | --- | --- | --- |
| **PPFP types** |  |  |  |  |  |
| PPIUD Insertion |  |  |  |  |  |
| Female sterilization |  |  |  |  |  |
| Male Sterilization |  |  |  |  |  |
| Interval IUD |  |  |  |  |  |
| Implant |  |  |  |  |  |
| Male condom |  |  |  |  |  |
| Injectable |  |  |  |  |  |
| Oral contraceptive pills |  |  |  |  |  |
